# Temporal relation between top-down and bottom-up processing in lexical tone perception

**DOI:** 10.3389/fnbeh.2014.00097

**Published:** 2014-03-25

**Authors:** Lan Shuai, Tao Gong

**Affiliations:** ^1^Department of Electrical and Computer Engineering, Johns Hopkins UniversityBaltimore, MD, USA; ^2^Department of Linguistics, University of Hong KongHong Kong, China

**Keywords:** lexical tone, ERP, lateralization, serial model, parallel model

## Abstract

Speech perception entails both top-down processing that relies primarily on language experience and bottom-up processing that depends mainly on instant auditory input. Previous models of speech perception often claim that bottom-up processing occurs in an early time window, whereas top-down processing takes place in a late time window after stimulus onset. In this paper, we evaluated the temporal relation of both types of processing in lexical tone perception. We conducted a series of event-related potential (ERP) experiments that recruited Mandarin participants and adopted three experimental paradigms, namely dichotic listening, lexical decision with phonological priming, and semantic violation. By systematically analyzing the lateralization patterns of the early and late ERP components that are observed in these experiments, we discovered that: auditory processing of pitch variations in tones, as a bottom-up effect, elicited greater right hemisphere activation; in contrast, linguistic processing of lexical tones, as a top-down effect, elicited greater left hemisphere activation. We also found that both types of processing co-occurred in both the early (around 200 ms) and late (around 300–500 ms) time windows, which supported a parallel model of lexical tone perception. Unlike the previous view that language processing is special and performed by dedicated neural circuitry, our study have elucidated that language processing can be decomposed into general cognitive functions (e.g., sensory and memory) and share neural resources with these functions.

## Introduction

Perception in general comprises two types of processing, bottom-up (or data-based) processing and top-down (or knowledge-based) processing, which are based, respectively, on incoming data and prior knowledge (Goldstein, 2009). For speech perception, the TRACE model (McClelland and Elman, 1986) claims that both types of processing are necessary, and the auditory sentence processing model (Friederici, 2002) proposes that the cognitive processes involved in speech perception proceed in a series of steps. Following these models, bottom-up processing such as acoustic processing of incoming signal and generalization of speech features happens first, whereas top-down processing such as recognition based on knowledge of phonemes, semantics, or syntax takes effect at a later stage of perception. These models, as well as other theories or models of speech perception (e.g., Liberman and Mattingly, 1985; Fowler, 1986; Stevens, 2002; Diehl et al., 2004; Hickok and Poeppel, 2007), are based primarily on evidence from non-tonal languages. Two issues remain to be explored: (a) whether the processing of tonal languages, which take up about 60–70% of world languages (Yip, 2002), follows the same cognitive processes; and (b) what is the role of lexical tone perception in a general model of speech perception. In addition, considering that the lexical tone attached to a syllable is carried mainly by the vowel nucleus of the syllable, the temporal dimension of cognitive processes underlying lexical tone perception is of special interest.

In this paper, we discussed the temporal relationship between bottom-up and top-down processing in lexical tone perception, with the purpose of not only examining the underlying mechanisms of lexical tone perception but also shedding valuable light on the general models of speech perception concerning tonal languages. Lexical tone is a primary use of pitch variations to distinguish lexical meanings (Wang, 1967). Noting that pitch perception belongs to the general auditory perception that is also shared by other animals (Hulse et al., 1984; Izumi, 2001; Yin et al., 2010) and word semantics are acquired primarily through language learning, lexical tone perception also entails both bottom-up and top-down processing. In our study, we defined *bottom-up processing* as auditory processing and feature extraction of incoming acoustic signals, which referred specifically to pitch contour perception. By contrast, we defined *top-down processing* as recognition and comprehension of incoming signals according to language knowledge, which referred specifically to influence of language experience on recognizing and comprehending a certain syllable in a tonal language. The recognition of a pitch contour as a certain tonal category was also ascribed to top-down processing.

Ample of available studies on lexical tone perception focused on the lateralization patterns of lexical tone processing (e.g., Van Lancker and Fromkin, 1973; Baudoin-Chial, 1986; Hsieh et al., 2001; Wang et al., 2001; Gandour et al., 2002, 2004; Tervaniemia and Hugdahl, 2003; Luo et al., 2006; Zatorre and Gandour, 2008; Li et al., 2010; Krishnan et al., 2011; Jia et al., 2013), and reported mixed results even under the same experimental paradigms. For example, by employing the dichotic listening (DL) paradigm and materials from Mandarin, Baudoin-Chial (1986) reported no hemisphere advantages of lexical tone perception, but Wang et al. (2001) found a left hemisphere advantage. Using fMRI, Gandour and colleagues compared lexical tone processing with intonation or vowel processing. The study of lexical tone and intonation (Gandour et al., 2003b) revealed a left hemisphere advantage in frontal lobe, whereas the study of lexical tone and segments (Li et al., 2010) discovered a right hemisphere advantage in fronto-parietal area for the perception of tones. A right lateralization of lexical tone perception was also reported in an ERP (Luo et al., 2006) and a DL experiment (Jia et al., 2013).

The inconsistent laterality effects could be due to different experimental conditions in these studies. For example, in the DL experiments reporting a left hemisphere advantage (Van Lancker and Fromkin, 1973; Wang et al., 2001), tonal language speakers participated into more difficult tasks than non-tonal language speakers, and the heavier load of these tasks (e.g., hearing trials at a faster pace) might enhance the left hemisphere advantage in tonal language speakers. By contrast, there were no hemisphere advantages in the study that had no task differences between tonal and non-tonal language speakers (Baudoin-Chial, 1986). In addition, in the DL tasks that involved meaningless syllables and hums, which could direct participants' attention toward pitch contours only, a right hemisphere advantage was shown (Jia et al., 2013). More importantly, whether language-related tasks are involved is the primary noticeable difference between studies showing an explicit right hemisphere advantage (e.g., Luo et al., 2006) and those reporting a left hemisphere advantage of lexical tone perception (Van Lancker and Fromkin, 1973; Hsieh et al., 2001; Wang et al., 2001; Gandour et al., 2002, 2004). For example, Luo et al. (2006) conducted a passive listening task in which participants were engaged in a silent movie, whereas the other studies carried out explicit language tasks such as lexical tone identification. Accordingly, the right lateralization reported in Luo et al. (2006)'s study could be attributed to the pure bottom-up effect without top-down influence, whereas the other studies did not address the underlying mechanisms of lexical tone perception. This could lead to the inconsistent results between these studies. These mixed results also reflect a multifaceted perspective on lexical tone processing and hemispheric lateralization. As stated in Zatorre and Gandour (2008)'s review, in tonal processing, “it appears that a more complete account will emerge from consideration of general sensory-motor and cognitive processes in addition to those associated with linguistic knowledge.”

To our knowledge, among the available studies, there was only one work (Luo et al., 2006) that discussed these two types of processing in lexical tone perception and a few that examined the cognitive processes involved for lexical tone perception (Ye and Connie, 1999; Schirmer et al., 2005; Liu et al., 2006; Tsang et al., 2010). In Luo et al. (2006)'s study, a serial model of lexical tone processing was proposed, which suggested that bottom-up processing (i.e., pitch perception) took effect in an early time window around 200 ms and top-down processing (i.e., semantic comprehension) happened in a late time window around 300–500 ms. The first half of this model was based on their experimental results that phonemes with slow- (lexical tone) and fast-changing (stop-consonant) acoustic properties inducted, respectively, right and left lateralization patterns of the MMN (Mismatch Negativity) component. The second half was proposed to address the confliction between their results and the previous literature that showed a general left hemisphere advantage of lexical tone perception. They proposed that during the late stage a left lateralization should be shown in the semantics-associated late ERP component, N400 (Kutas and Hillyard, 1980).

This serial model associated the right hemisphere advantage with bottom-up processing of lexical tones, and the left hemisphere advantage with top-down processing. In terms of lexical tone perception, there exists ample evidence in support of such association between the two types of processing and the two types of hemisphere advantage. For example, in studies of language experience and prosody, Gandour et al. (2004) dissociated linguistic processing in the left hemisphere and acoustic processing in the right hemisphere, by locating a left lateralization in certain brain regions in tonal language speakers and a right lateralization in non-tonal language speakers during speech prosody processing. Pitch processing has a right hemisphere advantage, as shown in behavior experiments such as DL (Sidtis, 1981), PET studies (Zatorre and Belin, 2001), and later fMRI studies (Boemio et al., 2005; Jamison et al., 2006); for review, see Zatorre et al. (2002). By contrast, compared to non-tonal language speakers, tonal language speakers have greater left hemisphere activities during lexical tone perception (Gandour et al., 1998, 2004; Hsieh et al., 2001; Wang et al., 2004), and multiple brain regions in the left hemisphere were believed to be the primary source of N400 (Lau et al., 2008). Noting these, we also adopted the lateralization pattern in our study to investigate top-down and bottom-up processing of lexical tones.

In addition, in Luo et al. (2006)'s study, there was insufficient direct evidence to manifest the top-down effect at the late stage of processing, because this study only explored acoustic factor without involving explicit language-related tasks or any linguistic factor. Therefore, it is hard to comprehensively evaluate Luo et al. (2006)'s serial model. Considering these, in order to make sure that language knowledge (top-down) would take effect, we adopted a number of explicit language-related tasks, including DL, lexical decision with phonological priming, and semantic violation. Meanwhile, we manipulated both the acoustic (requiring bottom-up processing) and semantic (requiring top-down processing) factors in the experimental design and analyzed the ERP components at both the early (around 200 ms) and late (around 300–500 ms) processing stages to explore the temporal relationship of the two types of processing during lexical tone perception.

Our experimental results showed that both bottom-up (acoustic) processing and top-down (semantic) processing exist in both the processing state around 200 ms and that around 300–500 ms, which inspired a parallel model of top-down and bottom-up processing in lexical tone perception. In the rest of the paper, we described the two ERP components traced in our experiments of Mandarin lexical tone perception (section ERP Components Reflecting Bottom-up and Top-down Processing), reported these experiments and their findings (section ERP Experiments of Lexical Tone Perception), discussed the lateralization patterns of the ERP components shown in these experiments and the derived parallel model of lexical tone perception (section General Discussions), connected language processing with general cognitive functions (section Language Processing and General Cognitive Functions), and finally, concluded the paper (section Conclusion).

## ERP components reflecting bottom-up and top-down processing

We examine two ERP components in our experiments, namely auditory P2 and auditory N400, which occur, respectively, in the early and late time windows after stimulus onset.

Auditory P2 is the second positive going ERP component. It usually has a central topographic distribution, and peaks in the early time window around 200 ms (Luck, 2005). The lateralization of P2 is subject to both acoustic properties and tasks (e.g., categorizing emotional words, Schapkin et al., 2000). The corresponding MEG component is P2m or M200. Previous research reported a general left lateralization of P2m in doing language-related tasks (e.g., perceiving consonants and vowels, Liebenthal et al., 2010), but acoustic properties of incoming signals also affect the lateralization of P2m (e.g., the voice onset time of consonants, Ackermann et al., 1999).

As a negative going potential, the auditory N400 appears in the late time window (around 250–550 ms) when the target sound stimulus is incongruent with the context (Kutas and Federmeier, 2011). The semantic violation paradigm can elicit N400 (Kutas and Hillyard, 1980). The phonological priming experiment can also elicit N400, when comparing the control condition with the priming condition (Praamstra and Stegeman, 1993; Dumay et al., 2001). The auditory N400 usually has a more frontal topological distribution than the visual N400 (Holcomb and Anderson, 1993; Kutas and Federmeier, 2011). In young population, the auditory N400 tends to have a frontal distribution (Curran et al., 1993; Tachibana et al., 2002). The source of N400 is believed to lie in the frontal and temporal brain areas (Maess et al., 2006; Lau et al., 2008), starting from 250 ms in the posterior half of the left superior temporal gyrus, migrating forward and ventrally to the left temporal lobe by 365 ms, and then moving to the right anterior temporal lobe and both frontal lobes after 370 ms (Kutas and Federmeier, 2011).

## ERP experiments of lexical tone perception

We designed three ERP experiments to explore the temporal relation of bottom-up and top-down processing in lexical tone perception. These experiments recruited Mandarin participants and traced the above two ERP components in three tasks, respectively, at the syllable, word, and sentence levels, which cover aspects of acoustics and phonetics, phonology, and semantics processing. According to the serial models (e.g., Friederici, 2002), these types of processing could be reflected by different ERP components shown at the early and late stages. However, a parallel model would predict a co-existence of these types of processing at both the early and late stages of lexical tone perception.

These experiments were designed primarily for the following two reasons. First, we were interested in clarifying whether top-down effects could happen at the early stage of a “lower-level” processing. To this purpose, we designed Experiment 1 using the DL task. Apart from the bottom-up effect on phoneme identification, we introduced a semantics factor to see whether a top-down effect inducted by this factor could exist in the early stage of perception and whether such effect could be reflected by the early ERP components (e.g., P2).

Second, we were interested in identifying bottom-up effects at the late stage of a “high-level” processing. To this purpose, we designed Experiment 2 using an auditory lexical decision task, which entailed a top-down semantic processing and a bottom-up processing induced by various types of phonological primes. We also designed Experiment 3 using a semantic violation task. In this task, semantic integration could be reflected by the late ERP component (e.g., N400). Meanwhile, phonemes bearing different acoustic properties could also induce the bottom-up acoustic processing at this stage.

Experiment 1 involved a DL task, which is a widely-adopted paradigm in behavioral and ERP studies examining the lateralization in the auditory modality. For example, Eichele et al. (2005) adopted a DL task using stop consonants as stimuli, and discovered that the latency of the ERP waveforms in the left hemisphere were shorter than those in the right hemisphere, thus reflecting a quicker response of the left hemisphere in perceiving stop consonants. Wioland et al. (1999) explored pitch perception in a DL task, and found that the ERP waveforms had higher amplitudes when the tone change happened in the left ear than in the right ear, thus indicating that the right hemisphere had prevalence in pitch discrimination. In our experiment, we adopted the DL paradigm to explore tone lateralization, and used the amplitude of auditory P2 as a temporal indicator, rather than ear advantages as in previous contradictory behavioral responses (Van Lancker and Fromkin, 1973; Baudoin-Chial, 1986), to reflect hemispheric specialization. We compared the lateralization patterns under tones and stop consonants in both words and non-words. In terms of acoustic properties, the stop consonants have fast-changing properties, whereas the lexical tones in Mandarin have slow-changing properties.

We expected an increase in the activity of the hemisphere for a certain processing, when there was a heavier load of information in the corresponding hemisphere. For example, in dichotic trials containing two different lexical tones, there would be a relatively greater right hemisphere advantage (equivalent to a less left hemisphere advantage) than dichotic trials containing two different stop-consonants but the same lexical tones. Similarly, dichotic trials containing words should generate a greater left hemisphere activity than dichotic trials containing non-words. In line with previous literature (Wioland et al., 1999; Luo et al., 2006), we examined the ERP waveforms in the C3 and C4 electrode groups.

Experiment 2 involved a lexical decision task with phonological priming. Priming refers to the phenomenon of acceleration in response after repetition. An early study of child language acquisition (Bonte and Blomert, 2004) adopted such a task. It used Dutch words and non-words as testing materials, and discovered different N400 reduction patterns in different language groups. Our experiment adopted a similar design, but used consonants and tones, as well as Chinese words and non-words as testing materials. In Experiment 2, consonant or tone primes appeared before target words, and we examined the auditory P2 and auditory N400 under the tone or consonant priming paradigm.

Other than the enhancement effect of DL as in Experiment 1, we expected that there would be a reduction of ERP components (smaller amplitude) due to the priming effect, and that semantic violation would induce a reduction of ERP amplitudes (as shown by the smaller amplitude in the positive component and greater amplitude in the negative component). These reductions could be greater in the corresponding hemisphere related to a certain processing. For example, the reduction caused by lexical tone priming should have a greater right hemisphere advantage (equivalent to a less left hemisphere advantage) compared to that of consonants, whereas the reduction caused by non-words should be greater in the left hemisphere compared to that of words. Considering the topographic distributions of auditory P2 and N400 (Curran et al., 1993; Tachibana et al., 2002; Luck, 2005) as well as the auditory brain regions involved in the tone priming tasks (Wong et al., 2008), In Experiment 2, we examined the ERP waveforms in the posterior (P3, P4) and frontal (F3, F4) electrode groups.

Experiment 3 involved a semantic violation task in sentences. N400 has been one of the most widely-explored ERP components in such studies, and we adopted the semantic violation paradigm to explore whether acoustic property affected the lateralization of auditory N400 occurring in the late time window during sentence comprehension, which was a high-level linguistic task. The violation was induced by changing either the stop consonant or the tone of the target syllable in a sentence. We expected a greater right lateralization of the N400 induced by lexical tone violation compared to consonant violation. Here we set the central (C3, C4) electrode groups as the regions of interest.

### Participants and settings

All these experiments were approved by the College Research Ethics Committee (CREC) of Hong Kong. Thirty-two university students (16 females, 16 males) volunteered for these experiments (age range: 19–29, mean = 27, *SD* = 4.2). In Experiment 1, data from all participants were analyzed. In Experiment 2, the data of one participant were excluded due to excessive eye movements, thus leaving 31 participants (age range: 19–29, mean = 25, *SD* = 2.3). In Experiment 3, the data of three participants were excluded, thus leaving 29 participants (age range: 19–29, mean = 26, *SD* = 2.8).

All these participants were native Mandarin speakers with no musical training. They had normal hearing (below 25 dBHL) in both ears and less than 10 dBHL differences at 125, 250, 500, 750, and 1000 Hz between the two ears, according to the PTA (pure tone analysis) test. They were all right-handed according to the Edinburgh handedness test (Oldfield, 1971), and reported no history of head damage or mental illness. They signed informed consent forms before each of these experiments, and got compensation at a rate of 50 HKD per hour after completing these experiments.

These experiments were conducted on three separate days. They were conducted in a dimly lit, quiet room. During experiment, participants were seated comfortably in front of a computer monitor, and the sound stimuli were presented via ER-3A air-conducting insert earphones, which diminished the environmental noise by 20–30 dB. Sound pressure level, measured by a sound level meter, was set to 75 dBSPL during experiment. The sound materials in these experiments were recorded from a female, native Mandarin speaker. The recording was conducted in a sound-proof booth using Shure SM10A microphone and Sony PCM-2700A audio recorder. The adjustments on recorded sound materials were implemented by the PSOLA (pitch-synchronous overlap add) algorithm in Praat (Boersma and Weenink, 2013), the experimental procedures were implemented using E-Prime (Psychology Software Tools, Pittsburgh, PA), and the statistical analyses were conducted using the SPSS software (version 18.0, SPSS Inc. Chicago, IL).

### EEG data recording and ERP processing

**Figure 1:**
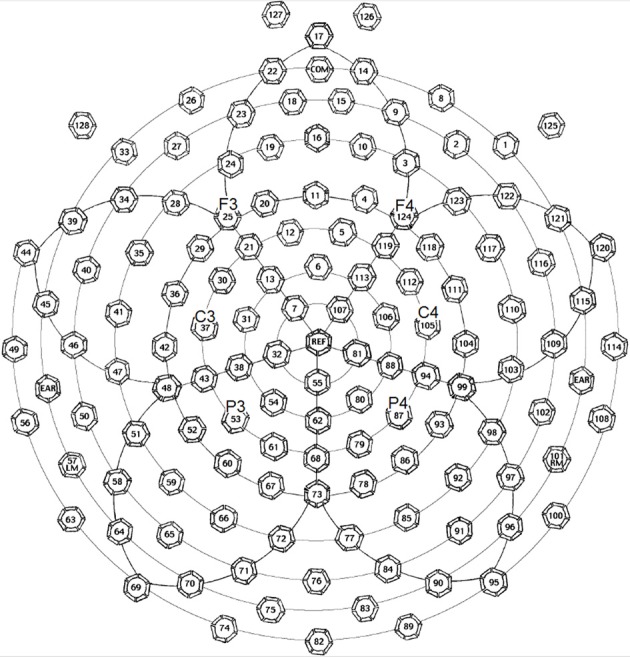
**Electrode positions of the EGI 128-channel Geodesic Sensor Net, in which the key electrodes (F3, F4, C3, C4, P3, and P4) for forming electrode groups to trace interested ERP waveforms and components are marked**. This figure is available at: ftp.egi.com/pub/documentation/placards/gsn200_128_map.pdf.

The EEG (electroencephalography) data were collected by a 128-channel EEG system with Geodesic Sensor Net (EGI Inc., Eugene, OR, USA) (see Figure 1). The impedances of all electrodes were kept below 50 kΩ at the beginning of the recording. In all the three experiments, participants were encouraged to avoid blinking or moving their body parts at certain points. Eye blinks and movements were monitored through electrodes located above and below each eye and outside of the outer canthi. The original reference point was the vertex. The ERPs were re-referenced to the averages of all 129 scalp channels in data processing (average reference). During recording, signals were sampled at 250 Hz with a 0.01–100 Hz band-pass filter.

During offline ERP processing, the recorded continuous data were filtered by a 40 Hz low-pass filter and segmented from −100 to 900 ms by referring to the stimulus onset. The segments having either an amplitude change exceeding 100 μV in the vertical eye channels and all electrodes, or a voltage fluctuation exceeding 50 μV in the horizontal eye channels were excluded from analyses. In each experiment, at least a half number of total trials were preserved for analysis in each condition and for every participant. The baseline correction was conducted from −100 to 0 ms.

In the following sections, we reported the materials, procedures, and results of these three experiments.

### Experiment 1: mandarin tone dichotic listening task

#### Materials

**Figure 2:**
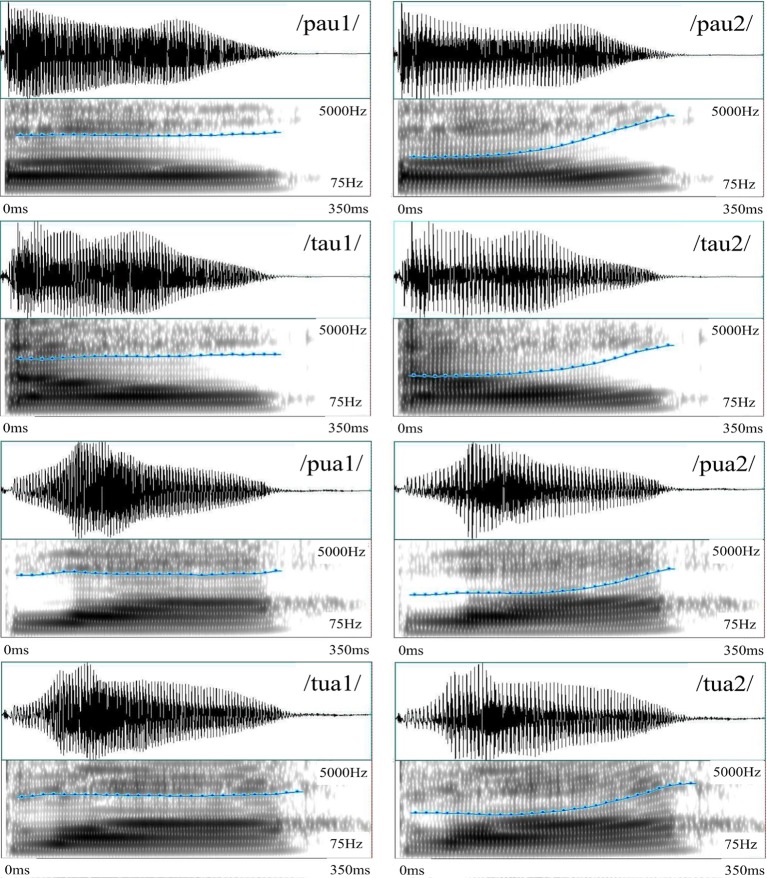
**Sound waveforms and spectrograms of the eight Mandarin syllables in Experiment 1**. The pitch contours of the syllables having level tones have stable level portions throughout the duration, whereas those of the syllables having rising tones start with a level portion (about half of the duration), followed by a rising portion (about half of the duration). The x-axis represents time (0–350 ms) and the y-axis represents frequency (75–5000 Hz) in spectrograms. The blue curve represents pitch contour at a different scale (50–500 Hz) superimposed on the spectrograms.

The recorded stimuli included Mandarin real- and pseudo-syllables, which are formed by two stop consonants (/p/ and /t/ in the IPA notation), two diphthongs (/au/ and /ua/ in the IPA notations), and two Mandarin tones (tone 1, the high level tone; and tone 2, the high rising tone). Eight syllables were constructed using these phonemes and tonemes, among which four were real-syllables, having corresponding Chinese characters, whereas the other four were pseudo-syllables, made of valid consonants and diphthongs but having no corresponding Chinese characters. All these stimuli were cut to 350 ms based on intensity profile. Figure 2 shows their waveforms and spectrograms, among which the pitch contours are also marked.

#### Procedure

In the DL task, participants simultaneously heard two distinct syllables, respectively, in their left and right ears, and were asked to respond, according to the Chinese character “
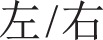
” (left/right) shown on the screen, both the consonant and the tone of the corresponding side of the auditory input, by pressing the corresponding keys on the respond pad.

We adopted a two-by-two design, with word and non-word as two levels of the lexicality factor, and stop consonant and tone as two levels of the acoustic contrast factor. The eight syllables formed four experimental conditions, including the word, consonant condition; word, tone condition; non-word, consonant condition; and non-word, tone condition, each containing two syllables (see examples in Table 1). In the two word conditions, the words were formed by meaningful real-syllables in Chinese; in the two non-word conditions, the non-words were formed by pseudo-syllables having no meanings in Chinese. In the two consonant conditions, the two syllables had the same diphthong and tone, but different initial consonants; in the two tone conditions, however, the two syllables had the same initial consonant and diphthong, but different tones. To balance the two syllables, respectively, played to the left and right ears of participants and the two directions in participants' responses (left or right), each of these four conditions corresponded to four DL trials. In total, there were 16 DL trials.

**Table 1:**
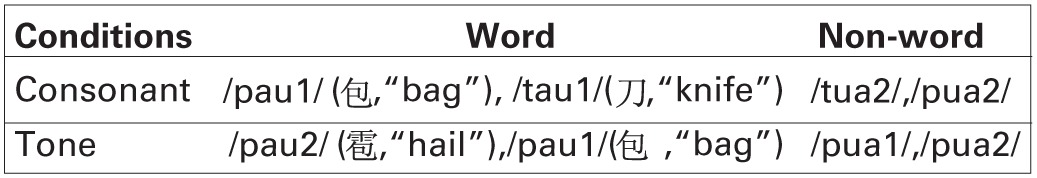
**Experimental conditions in Experiment 1, each containing two syllables and four DL trials**.

*Words have corresponding Chinese characters (shown in brackets, together with their meanings). The pronunciations are annotated with the IPA characters, the numbers in which denote Mandarin tones*.

In each trial, a fixation first appeared on the center of the screen and remained there. After 400 ms, the two syllables in a DL trial were simultaneously played to the left and the right ears of participants, respectively. Participants were encouraged not to blink or move their body parts during the appearance of the fixation. After 1000 ms, the fixation on the screen was replaced by the Chinese character that indicated left or right, and accordingly, participants reported the consonant and the tone of the syllable heard by their corresponding ears. The purpose of letting participants hear the stimuli before seeing the indication (left or right) was to avoid inducing prior bias in their attention. The indication stayed on the screen for 2000 ms, during which participants gave their responses. The presentation sequence of the stimuli was randomized, and the order of choices between the two consonants and between the two tones on the response box was counter-balanced across participants.

Participants first went through a practice session (16 trials) to familiarize the experimental paradigm. In the experimental session, a total of 256 trials were presented to participants, each lasting around 5 s. The experiment consisted of four blocks, each having 64 trials that lasted about 5 min. In each block, the 16 DL trials randomly repeat four times. Participants could take a 2-min break after each block, and the whole experiment lasted approximately 30 min.

#### Data analysis and results

As for the behavioral data, the overall rate of response was 95.7%. A Three-Way repeated-measures ANOVA of rates of correct response, with lexicality (word vs. non-word), acoustic contrast (consonant vs. tone), and hemisphere (left vs. right) as three factors, revealed a significant three-way interaction [*F*_(1, 31)_ = 5.981, *p* < 0.024, η^2^_*p*_ = 0.239]. In addition, the *post-hoc* analysis revealed a significant left hemisphere (right ear) advantage in the non-word, consonant condition [*t*_(19)_ = −2.280, *p* < 0.034]. Since participants needed to respond to both the consonant and the tone of the syllable in one ear, we did not analyze the reaction time.

As for the ERP data, considering the central distribution of auditory P2 (Luck, 2005) and previous literature (Luo et al., 2006), we averaged the data recorded by the four homolog pairs of adjacent central electrodes including C3 and C4 [electrodes 37 (C3), 38, 42, 43 in the left hemisphere, and 105 (C4), 88, 104, 94 in the right hemisphere, according to the EGI system] for analysis. Since the P2 peak appeared between 180 and 200 ms, we averaged the amplitude of P2 within this time range. A Three-Way repeated-measures ANOVA of P2 amplitudes, with lexicality, acoustic contrast, and hemisphere as three factors, revealed two significant interactions, one between acoustic contrast and hemisphere [*F*_(1, 31)_ = 7.744, *p* < 0.0091, η^2^_*p*_ = 0.200] and the other between lexicality and hemisphere [*F*_(1, 31)_ = 12.687, *p* < 0.0012, η^2^_*p*_ = 0.290], and two main effects, hemisphere [*F*_(1, 31)_ = 14.393, *p* < 0.0006, η^2^_*p*_ = 0.317] and acoustic contrast [*F*_(1, 31)_ = 22.024, < 0.0001, η^2^_*p*_ = 0.415].

**Figure 3:**
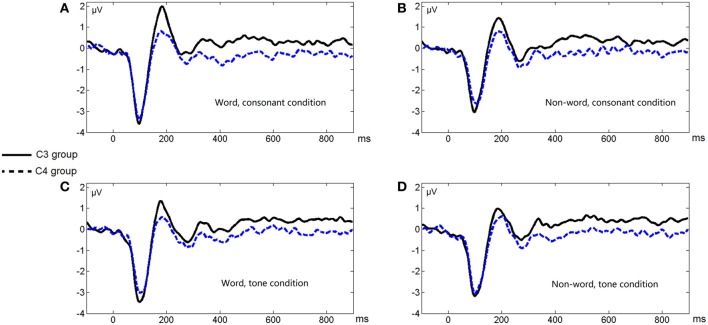
**Average ERP waveforms of the C3 and C4 electrode groups under the four conditions of Experiment 1**. **(A)** Word, consonant condition; **(B)** Non-word, consonant condition; **(C)** Word, tone condition; **(D)** Non-word, tone condition.

**Figure 4:**
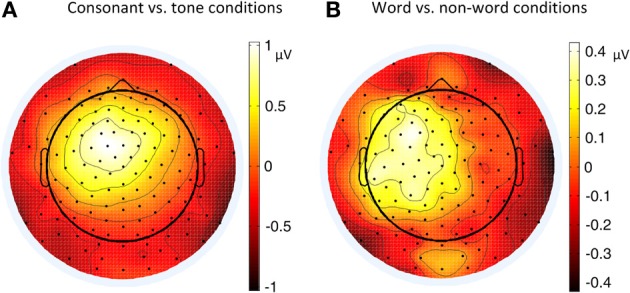
**Topographies of P2 (180–200 ms) contrasts in different conditions of Experiment 1**. **(A)** Consonant vs. tone condition; **(B)** Word vs. non-word condition.

**Figure 5:**
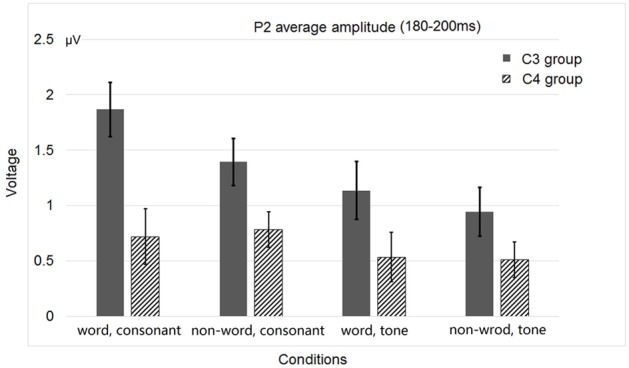
**Average P2 amplitudes of the C3 and C4 electrode groups in Experiment 1**.

Figure 3 shows the average ERP waveforms of the C3 and C4 electrode groups, Figure 4 shows the topographies of the ERP component contrasts, and Figure 5 shows the average P2 amplitudes between 180 and 200 ms in different conditions.

The greater left lateralization of P2 shown by comparing the word conditions with the non-word conditions reflected top-down processing in the early time window around 200 ms. This indicates the involvement of language experience in the process. In order to differentiate words from non-words, participants needed prior language knowledge, which casted as a top-down effect, regardless of whether this effect belonged to word form recognition (Friederici, 2002) or semantic processing.

The greater left lateralization of P2 shown by comparing the consonant conditions with the tone conditions also reflected bottom-up processing in the same time window. The difference between these conditions was the speed of changes in acoustic cues. In line with previous results (Jamison et al., 2006), we found a greater left lateralization in perceiving fast changing acoustic cues (formant transition in stop consonants) and a less left lateralization in perceiving relatively-slow changing acoustic cues (pitch changes in tones). We expected that the less left lateralization of tone processing compared to consonant processing was due to the greater right lateralization of tone processing compared to consonant and rhyme in certain brain regions in Li et al.'s work 2010.

### Experiment 2: lexical decision task with phonological priming

#### Materials

The recorded stimuli included monosyllabic words as primes and disyllabic words as targets. These words could be real- or non-words in Chinese. The mean duration of the primes was 383.06 ms (range: 251–591 ms, *SD* = 49.17), and that of the targets 619.58 ms (range: 510–751 ms, *SD* = 52.05). There was no significant differences of either the prime duration [*F*_(5, 354)_ = 1.098, *p* = 0.3609, η^2^_*p*_ = 0.015] or the target duration [*F*_(5, 354)_ = 1.244, *p* = 0.2878, η^2^_*p*_ = 0.017] between conditions. The onset asynchrony between the primes and the targets was fixed at 1000 ms. The sound intensity of the primes was set to 55 dB, and that of the targets 75 dB. The purpose of presenting primes at a lower intensity was to maximize the priming effect (Lau and Passingham, 2007).

#### Procedure

In the lexical decision task, participants were asked to judge whether the heard disyllabic words (targets) were words or non-words. They were instructed to ignore the monosyllabic words (primes) played before the disyllabic words and focus on the latter.

Similar to Experiment 1, we adopted a two-by-two design, with word and non-word as the two levels of the lexicality factor, and stop consonant and tone as the two levels of the priming condition factor. The materials in Table 2 formed six experimental conditions. In the two consonant conditions, the syllable in the prime shared the initial consonant with the first syllable of the target; in the two tone conditions, the syllable in the prime shared the tone with the first syllable of the target; and in the two control conditions, the syllable in the prime and the first syllable of the target shared no phonemes or tonemes.

**Table 2:**
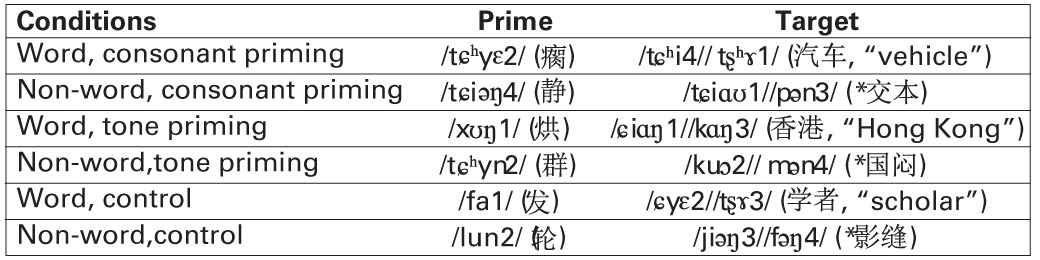
**Example materials and experimental conditions in Experiment 2**.

The pronunciations are annotated with the IPA characters, the numbers in which denote Mandarin tones. As for the primes, Chinese characters are shown in brackets. As for the targets, the Chinese words are shown in brackets, together with their meanings. Each non-word includes two real-syllables (shown in brackets), but their combination does not form a meaningful disyllabic word in Chinese (marked by ^*^).

In each trial, participants first heard the prime. After 600 ms, a fixation appeared on the center of the screen and remained there. After another 400 ms, participants heard the target. After another 1000 ms, the fixation disappeared, and participants had 3000 ms to give their response, by pressing one of the two keys marked by “yes” and “no” in the keyboard. Participants were encouraged not to blink or move their body parts during the appearance of the fixation, and not to respond until the fixation disappeared. One half of the participants responded to the “yes” key with their left index finger and the “no” key with their right index finger. The other half did the reverse. The left or right response order was randomly assigned to participants.

There were in total 360 trials, with 60 trials in each of the six conditions. Each trial lasted around 5 s. Participants first went through a practice session (36 trials) to familiarize the experimental paradigm. The experiment consisted of six blocks, each having 60 trials and lasting about 5 min. Trials were arranged in a random order. Participants could take a 2-min break after each block, and the whole experiment lasted approximately 40 min.

#### Data analysis and results

As for the behavioral data, the response correctness was 96.0%. The average reaction time was 962.72 ms (*SD* = 159.99). Outliers greater than three times of standard deviation from mean were replaced with mean value in each participant. A marginal significant priming effect in the word, consonant priming condition was observed [*t*_(30)_ = −1.993, *p* < 0.055], while the tone priming conditions showed interference effects [as for the word, tone priming condition, *t*_(30)_ = 1.822, *p* = 0.078; as for the non-word, tone priming condition, *t*_(30)_ = 2.524, *p* < 0.017].

As for the ERP data, considering both of the P2 topography (Luck, 2005) and the brain regions for tone priming (Wong et al., 2008), we averaged four homolog pairs of adjacent posterior electrodes including P3 and P4 [electrodes 53 (P3), 61, 54, 38 in the left hemisphere, and 87 (P4), 79, 80, 88 in the right hemisphere, according to the EGI system] for analysis. We calculated the priming effects of tones or consonants by subtracting the ERP waveforms in the word or non-word control conditions from those in the word or non-word experimental conditions. Similar to Experiment 1, based on a Three-Way repeated-measures ANOVA, with lexicality (word vs. non-word), priming condition (consonant vs. tone), and hemisphere (left vs. right) as three factors, we found that the average P2 amplitude in the early time window (200–220 ms, P2 peak values were within this time range) showed two significant interactions, one between lexicality and hemisphere [*F*_(1, 30)_ = 5.618, *p* < 0.0244, η^2^_*p*_ = 0.158], and the other between priming condition and hemisphere [*F*_(1, 30)_ = 8.515, *p* < 0.0066, η^2^_*p*_ = 0.221], and a main effect of lexicality [*F*_(1, 30)_ = 8.242, *p* < 0.0074, η^2^_*p*_ = 0.216]. Similar to Experiment 1, these results indicated that both semantics and acoustic properties affected lateralization.

Apart from P2, we conducted another analysis of the ERP waveform in the late time window (500–550 ms) based on four homolog pairs of adjacent frontal electrodes including F3 and F4 [electrodes 25 (F3), 28, 29, 35 in the left hemisphere, and 124 (F4), 123, 118, 117 in the right hemisphere, according to the EGI system]. Rather than deriving auditory N400 by contrasting non-word and word conditions, we analyzed these conditions separately in order to preserve the lexicality factor and make factors in statistic analysis consistent with the previous one based on P2, though the interested time windows in these two analyses were different. The data for statistical analysis here were all from priming conditions without subtracting control conditions. By examining the same three factors as in the previous analysis, this analysis showed three main effects, priming condition [*F*_(1, 30)_ = 6.564, *p* < 0.0157, η^2^_*p*_ = 0.180], lexicality [*F*_(1, 30)_ = 7.892, *p* < 0.0087, η^2^_*p*_ = 0.208], and hemisphere [*F*_(1, 30)_ = 9.193, *p* < 0.0050, η^2^_*p*_ = 0.235]. Priming condition interact significantly with lexicality [*F*_(1, 30)_ = 5.636, *p* < 0.0242, η^2^_*p*_ = 0.158]. More importantly, there was a significant interactions between lexicality and hemisphere [*F*_(1, 30)_ = 10.729, *p* < 0.0027, η^2^_*p*_ = 0.263].

**Figure 6:**
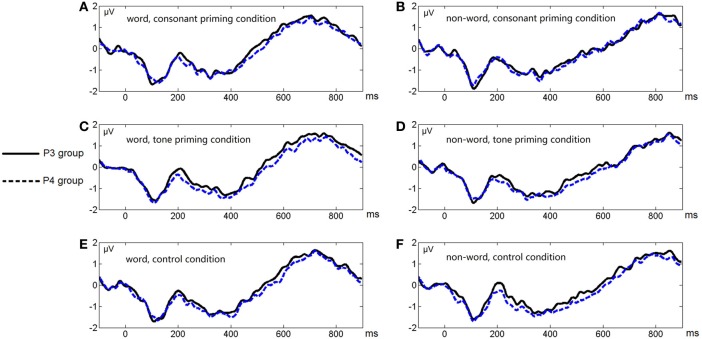
**Average ERP waveforms of the P3 and P4 electrode groups under the six conditions of Experiment 2**. **(A)** Word, consonant priming condition; **(B)** Non-word, consonant priming condition; **(C)** Word, tone priming condition; **(D)** Non-word, tone priming condition; **(E)** Word, control condition; **(F)** Non-word, control condition.

**Figure 7:**
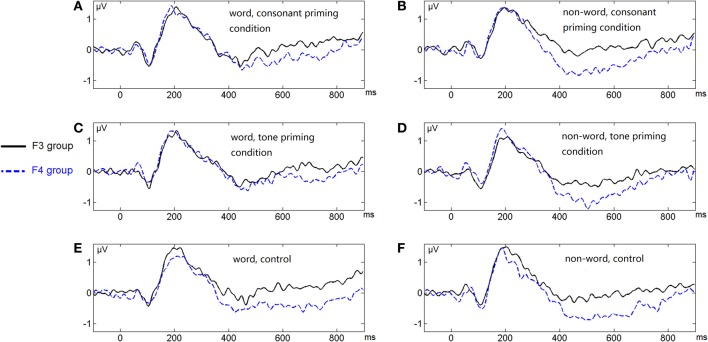
**Average ERP waveforms of the F3 and F4 electrode groups under the six conditions of Experiment 2**. **(A)** Word, consonant priming condition; **(B)** Non-word, consonant priming condition; **(C)** Word, tone priming condition; **(D)** Non-word, tone priming condition; **(E)** Word, control condition; **(F)** Non-word, control condition.

**Figure 8:**
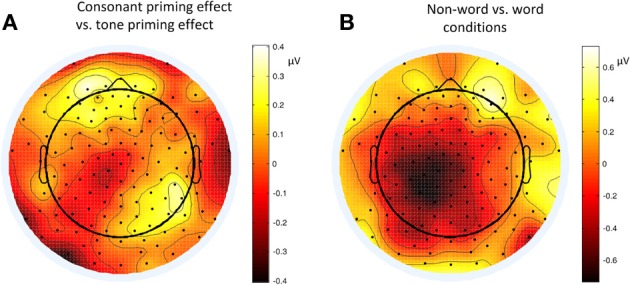
**Topographies of P2 (200–220 ms) contrasts in different conditions of Experiment 2**. **(A)** Consonant priming effect vs. tone priming effect; **(B)** Non-word vs. word condition.

**Figure 9:**
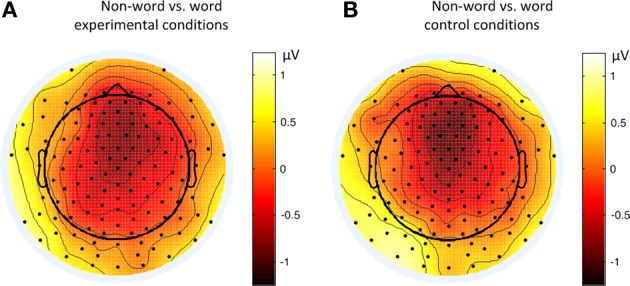
**Topographies of the contrasts of the late ERP component (500–550 ms) in different conditions of Experiment 2**. **(A)** Non-word vs. word experimental conditions; **(B)** Non-word vs. word control conditions.

**Figure 10:**
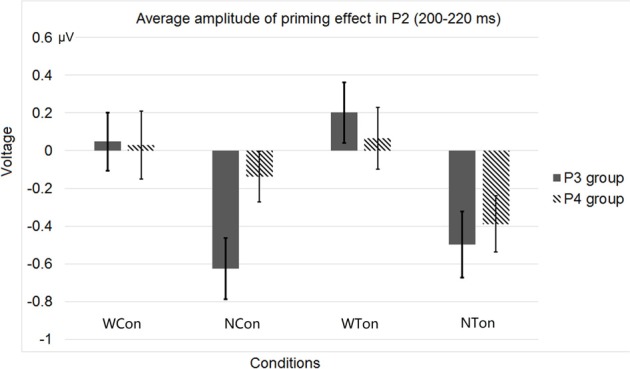
**Average P2 amplitudes within 200 and 220 ms in the P3 and P4 electrode groups in Experiment 2**. “WCon” denotes the comparison between the word, consonant priming condition and the word, control condition, and “NCon” between the non-word, consonant priming condition and the non-word, control condition, “WTon” between the word, tone priming condition and the word, control condition, “NTon” between the non-word, tone priming condition and the non-word, control condition.

**Figure 11:**
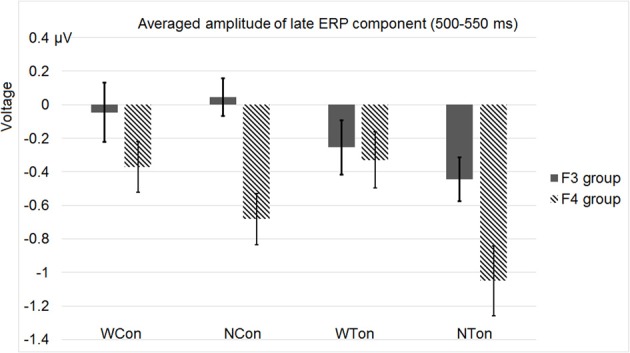
**Average amplitudes of the late ERP component around 500–550 ms in the F3 and F4 electrode groups in Experiment 2**.

Figure 6 shows the average ERP waveforms of the P3 and P4 electrode groups, and Figure 7 shows those of the F3 and F4 electrode groups. Figure 8 shows the topographies of the contrasts of the P2 component around 200 ms (200–220 ms), and Figure 9 shows those of the late ERP component around 500 ms (500–550 ms). Figure 10 shows the average amplitudes of P2, and Figure 11 shows those of the late component in different conditions.

The lateralization pattern of P2 could be interpreted as follows. The greater the priming effect, the lower the amplitude of P2, due to the repetition effect. Since there was no main effect of priming condition, the priming effects of consonants and tones were not much different from each other. However, the left and right hemispheres showed different trends of these priming effects, due to the significant interaction between hemisphere and priming condition. By examining the amplitude difference between the priming effects of consonants and tones (see Figure 8A) and those of words and non-words (see Figure 8B), we found a stronger priming effect of consonants than tones was shown in the left hemisphere compared to the right hemisphere around centro-parietal region, and a greater left hemisphere advantage in processing words compared to non-words. These showed that the left hemisphere responded significantly differently in the consonant priming and tone priming conditions, as well as word and non-word conditions. In line with Experiment 1, these results illustrated that both bottom-up and top-down processing took place around 200 ms. The left hemisphere responded to the fast changing acoustic cues greater than the slow changing acoustic cues, and it also responded to word semantics greater than non-words that had no meanings.

By comparing the word and non-word conditions, we found that the amplitudes of frontal region at the late component (around 500 ms) were lower in the non-word conditions compared to the word conditions (consistent with the main effect of lexicality), which was right lateralized (consistent with the interaction between lexicality and hemisphere) (see Figure 9). Such a right lateralization was also shown in Experiment 3 when comparing the tone-induced N400 with the consonant-induced N400.

### Experiment 3: semantic violation in sentences

#### Materials

The recorded stimuli included a number of Chinese sentences. Each sentence consisted of 11 syllables, and the last two were always a verb and its object. Semantic violation was induced by changing the tone or consonant of the last syllable of a sentence. The average duration of these sentences from the onset of the first syllable to the stop of the last one was 3206.1 ms (*SD* = 156.1). There was no significant difference of these durations between different conditions [*F*_(2, 177)_ = 0.697, *p* = 0.4996, η^2^_*p*_ = 0.008]. The intensity of these sentences was adjusted to 75 dB. Table 3 shows examples of such sentences.

**Table 3:**
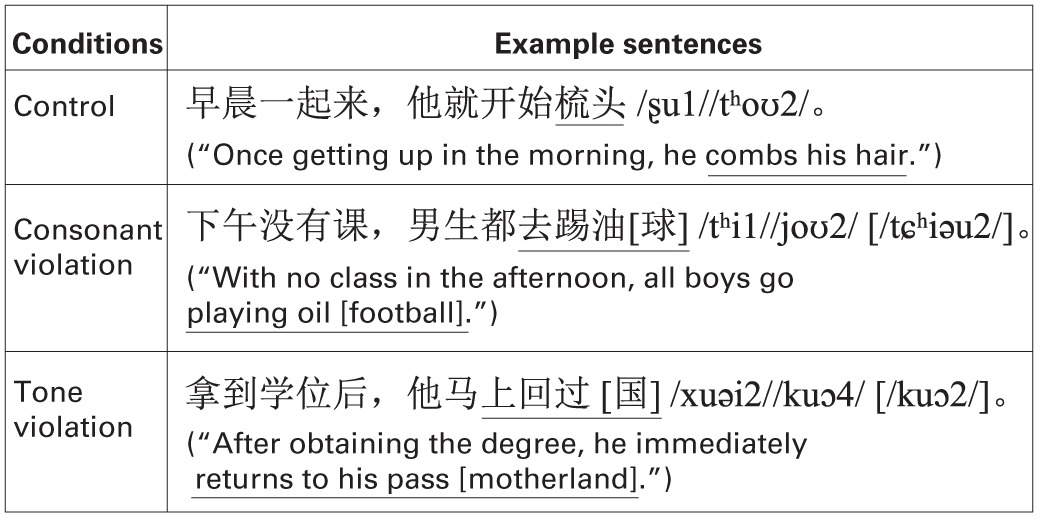
**Example sentences and experimental conditions in Experiment 3**.

*The last syllable of each sentence is the target word, and all the previous ones are the context. In each condition, the Chinese transcription of the sentence and its meaning are shown. The pronunciations of the last two syllables are annotated with the IPA characters, the numbers in which denote Mandarin tones. In the violation conditions, the last syllable induces violation, and the syllable after change is still a real-syllable in Chinese. For comparison, the syllables within the square brackets are consistent with the context*.

#### Procedure

In the sentence comprehension task, participants were asked to judge whether the last syllable in a sentence was consistent with the context or not. Since the violation appeared toward the end of the sentence, participants were encouraged not to blink or move their body parts toward the end of the sentences.

There were three experimental conditions (see Table 3): in the control condition, there was no semantic violation; in the consonant violation condition, the violation was induced by changing the initial of the last syllable of the sentence; and in the tone violation condition, the violation was induced by changing the tone of the last syllable of the sentence.

In each trial, a fixation first appeared on the screen and remained there. After 400 ms, one of the sentences was presented to participants. The fixation disappeared at 1000 ms after the onset of the last syllable in the sentence, and then, participants had 2000 ms for response, by pressing one of the two keys marked by “yes” and “no” in the keyboard. One half of the participants pressed the “yes” key with their left index finger and the “no” key with their right index finger. The other half did the reverse. The left or right response order was randomly assigned to participants.

There were in total 180 testing sentences, with 60 sentences in each condition. We also added ten filler sentences, each having the same length as the testing sentences, no semantic violation, and a free structure. The purpose of incorporating filler sentences was to make the yes and no responses have equal chances. The average length of each trial was 6606.1 ms (*SD* = 156.1). Participants first went through a practice session (30 trials) to familiarize the experimental paradigm. The experiment consisted of six blocks, each containing 40 sentences. These sentences included ten randomly chosen sentences from each of the three conditions, and ten filler sentences. The order of these sentences was randomized. Each block lasted about 4 min. Participants could take a 2-min break between blocks. The whole experiment lasted about 30 min.

#### Data analysis and results

As for the behavioral data, the response correctness was 94.3%. The averaged reaction time was 854.13 ms (*SD* = 186.61). Outliers greater than three times of standard deviation from mean were replaced by the mean value in each participant.

As for the ERP data, we referred to the data recorded by the four homolog pairs of adjacent electrodes including C3 and C4 [electrodes 37 (C3), 38, 42, 43 in the left hemisphere, and 105 (C4), 88, 104, 94 in the right hemisphere, according to the EGI system] for analysis. A Two-Way repeated-measures ANOVA, with violation type (consonant vs. tone) and hemisphere (left vs. right) as two factors, revealed a main effect of violation type [*F*_(1, 28)_ = 9.622, *p* < 0.0044, η^2^_*p*_ = 0.256], and a significant interaction between hemisphere and violation type [*F*_(1, 28)_ = 9.573, *p* < 0.0044, η^2^_*p*_ = 0.255]. A *post-hoc T*-test revealed a significance of the right lateralized N400 [*t*_(28)_ = 2.164, *p* < 0.0391] in the tone violation condition, and no significant lateralization in the consonant violation condition. Similar to Experiment 2, this analysis considered the control conditions, by subtracting the ERP waveforms in them from those in the experimental conditions.

**Figure 12:**
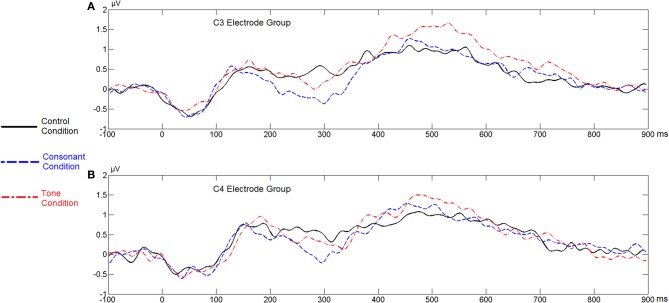
**Average ERP waveforms of the F3 and F4 electrode groups in Experiment 3**. **(A)** C3 electrode group; **(B)** C4 electrode group.

**Figure 13:**
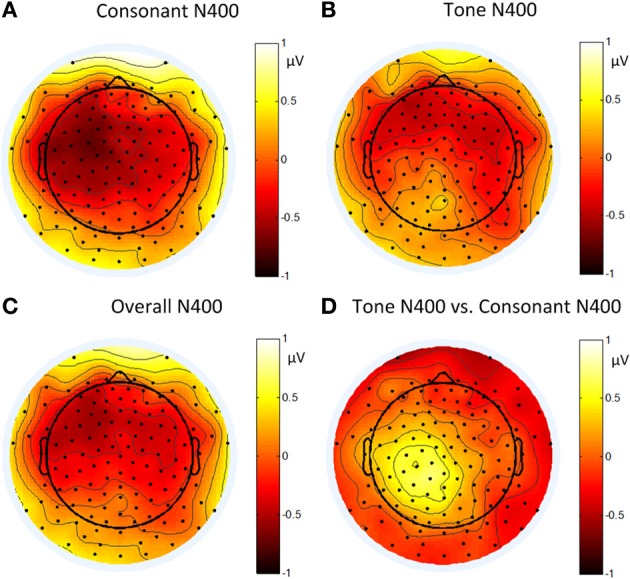
**Topographies of: **(A)** the N400 induced by consonant violation; **(B)** the N400 induced by tone violation; **(C)** the N400 induced by summing up consonant and tone violation; and **(D)** the difference between the N400 in **(B)** and **(A)** in Experiment 3**.

**Figure 14:**
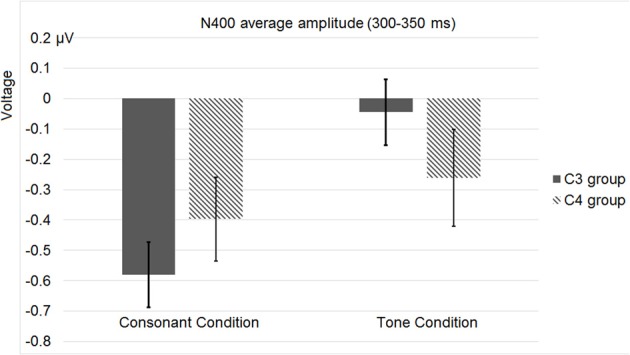
**Average N400 (300–350 ms) amplitudes of the C3 and C4 electrode groups in Experiment 3**. “Consonant” and “tone” denote the consonant and tone violation conditions.

Figure 12 shows the average ERP waveforms of the C3 and C4 electrode groups, Figure 13 shows the topographies and differences of auditory N400, and Figure 14 shows the average amplitudes of N400 (300–350 ms) in different conditions.

The significant interaction between hemisphere and violation type reflected bottom-up processing. Noticeably, there was a right lateralization in the difference between the N400 inducted by tone violation and that induced by consonant violation, which supported that bottom-up (acoustic) processing also existed in the late stage of perception.

## General discussions

To sum up, in Experiment 1 and Experiment 2, we discovered both top-down (semantic) and bottom-up (acoustic) processing in the early time window around 200 ms. In the late stage around 300–500 ms, we only found the top-down effect in Experiment 2, probably because that the phonological primes were presented too early and the bottom-up effect could not last long enough. However, in Experiment 3, the bottom-up effect was reflected by N400 in the late stage. As indicated by the late component in Experiment 2 and Experiment 3, we suggested that both top-down and bottom-up processing existed at the late stage. The N400 component had a shorter latency in Experiment 3 than that in Experiment 2 because of the context effect, and the topography of the earlier N400 in Experiment 3 had a more central distribution compared to the late frontal N400 in Experiment 2, which is consistent to the description of N400 in time and spatial domains (Kutas and Federmeier, 2011).

### Relation between top-down and bottom-up processing during lexical tone perception

During lexical tone perception, the prior knowledge formed by language experience helps match a large variety of pitch contours onto clear tonal categories, and the semantic representation requires combining tonal categories with carrying syllables. Therefore, the prior knowledge of tonal categories and the lexical semantics of linking tonal categories with carrying syllables become the primary top-down factors during lexical tone perception. Since semantic and categorical information of phonemes is processed dominantly in the left hemisphere (McDermotta et al., 2003; Liebentral et al., 2005), a general left lateralization pattern during lexical tone perception reflects top-down processing. Similarly, the primary acoustic cue of lexical tone is pitch variation, and processing of pitch variations is bottom-up. Since it is widely accepted that the right hemisphere is dominant for pitch processing (Sidtis, 1981; Tenke et al., 1993), a relative right lateralization pattern during lexical tone perception also reflects bottom-up processing.

By tracing the auditory ERP components in the early and late time windows, our experiments explore the general and relative lateralization patterns in conditions with or without lexical semantics and slow or fast changing acoustic cues. Though involving distinct tasks, these experiments reveal two consistent lateralization patterns of early (P2) and late (N400) ERP components: (a) manipulation of linguistic information in words modulates lateralization: meaningful words tend to generate a greater left lateralization; and (b) manipulation of physical property of auditory input modulates lateralization: faster changing cues generate a greater left lateralization. These two patterns reflect top-down (lexical semantic) and bottom-up (acoustic phonetic) processing, respectively. Since lexical tone perception concerns both acoustic properties and lexical semantics, the observed lateralization patterns are never a simple dichotomy of purely left or right lateralization, as observed in the previous studies focusing only on one aspect of lexical tone perception. The modulation effects on lateralization at both the early and late time windows suggest that both top-down and bottom-up processing exist at different stages of perception, which support a parallel model of top-down and bottom-up processing.

### Three-stage, parallel lexical tone processing model

Previous explorations revealed that lexical tone differed from segmental cues (Ye and Connie, 1999; Lee, 2007), and that lateralization of lexical tone processing differed from that of segment processing (Li et al., 2010). Neuroimaging studies of lexical tone processing also revealed that separate brain regions were involved in perceiving lexical tones compared to segments (Gandour et al., 2003a). Apart from perception, differences between tone and segment processing were also found in lexical tone production (Liu et al., 2009). However, all these explorations did not disentangle language experience as top-down factors and acoustic cues as bottom-up factors. Treating processing of pitch information and semantic role as a cohort processing makes it difficult to figure out the cognitive processes during tone processing (Zatorre and Gandour, 2008), since lexical tone processing involves both acoustic and linguistic factors.

In our study, we regard pitch processing as bottom-up processing in lexical tone perception, since it concerns acoustic cues, and semantic processing as top-down processing, since it involves language experience. In this way, we separate these two cognitive functions called for tone perception. By exploring the temporal relation between bottom-up and top-down processing in the time windows around 200 ms and around 300–500 ms after stimulus onset, we confirm that both types of processing participate in tone perception during these early and late time periods.

Apart from these findings, there was also evidence showing a greater left lateralization of contour tones than level tones as well as a general left lateralization of Cantonese lexical tone perception in the N1 component around 100 ms after stimulus onset (Ho, 2010; Shuai et al., in press). The result of Cantonese perception reflected a bottom-up acoustic effect at around 100 ms, whereas the general left lateralization was consistent with the lateralization of top-down effect as observed in our experiments of Mandarin tone perception.

Based on the findings in our experiments and those previous studies, we propose a detailed, three-stage, parallel model of lexical tone processing. The three stages are defined based on the occurrences of different ERP components in our and previous experiments (e.g., N1, around 100 ms after stimulus onset; P2, around 100–300 ms; and N400, after 300 ms; among these components, N1 and P2 belong to the early stage and N400 belongs to the late stage).

At the first stage (before and around 100 ms after stimulus onset, as in Ho, 2010 and Shuai et al., in press), syllable initials are processed to provide the basic structure of the syllable. At this stage, if the syllable starts with a vowel or a sonorant consonant, pitch information is available; if it starts with a voiceless consonant, there is no pitch information. In either case, tonal category is not formed yet, since the recognition of pitch variation or pattern, as slow-varying acoustic cues, requires a time window longer than 100 ms. At this stage, top-down linguistic processing also occurs, no matter whether there is contextual information before the syllable initial. With contextual information, top-down processing would become stronger, though it may play distinct roles from bottom-up processing at this stage.

At the second stage (100–300 ms after stimulus onset, as in our experimental conditions), predictions about pitch patterns and following segmental information are made, based on the information gathered at the first stage and prior language experience. At this stage, semantic information at a gross level is also activated via top-down processing, which is initiated based on the information gathered at the first stage. Since lexical tone is not fully recognized, bottom-up processing is still ongoing. According to previous literature (Kaan et al., 2007, 2008), pitch variation in the middle proportion of a syllable is the most important for recognizing contour tones for native tonal language speakers. At this stage, listeners keep integrating the incoming pitch information with the information gathered at the first stage in order to recognize the tone and the whole syllable. In this sense, both top-down prediction of tonal categories and bottom-up generalization of tonal categories are taking places at this stage.

At the third stage (after 300 ms, as in our experimental conditions), top-down processing becomes more prominent, helping listeners recognize the tone, the whole syllable, and its meaning, based on prior or previous language experience and the information gathered at the first two stages. Detailed semantic information is recognized at this stage. However, bottom-up processing keeps taking effect, helping to confirm the recognized tone and syllable.

Compatible with our findings in the series of experiments involving various levels of language processing, this parallel model of lexical tone perception can shed important lights on general speech perception models in many aspects.

First, this parallel model, as a cognitive model, proposes that top-down processing is available in both the early and the later stages of lexical tone perception, especially when contextual information is available.

Second, this parallel model refutes the claim that semantic processing (top-down) occurs always after acoustic processing (bottom-up), as in Friederici's general auditory processing model and Luo et al.'s serial model. The influence of previous language experience always exists during speech perception, especially in the case of auditory sentence processing. There are various types of cues and ample information that can serve as context for perceiving incoming syllables, and the neural system in humans always makes predictions. Even in the case of single syllable perception, if the task is linguistic relevant, top-down processing based on language experience is inevitable. As shown in our experiences, the greater left lateralization of P2 and N400 under the semantic conditions explicitly reflects such language-relevant, top-down processing.

Third, the bilateral lexical tone processing also complements to the neuroimaging models of speech perception. For example, in the dorsal-ventral pathway hypothesis of speech perception (Hickok and Poeppel, 2007), phoneme perception was regarded as involving only the left hemisphere, since such generalization did not involve tonal languages. Considering that 60–70% of world languages are tonal languages (Yip, 2002), a speech perception model leaving out tones is incomplete.

### Top-down processing at the preattentive stage

Humans often predict incoming signals based on experience. Therefore, top-down processing could accompany the whole processes of speech perception. In addition, information generated by bottom-up processing is also used to match the prediction coming from top-down processing. In this sense, top-down processing is a pre-determined process, making the relevant hemisphere or brain regions get prepared for the forthcoming task. When stimuli come in, they will evoke responses from the corresponding hemisphere or brain regions, and these responses may adjust or even alter the degrees of lateralization. A similar effect is shown in the attention or memory modulation of lateralization in DL tasks (Hugdahl, 2005; Saetrevik and Hugdahl, 2007). For example, by asking participants to attend to stimuli in either left or right ears (Hugdahl, 2005), the degree of lateralization is adjusted in favor of the side attended.

Even though long-term language experience keeps affecting automatic processing at the preattentive stage, it is generally hard to observe online top-down processing at this stage. It is only until recently that the automatic top-down processing has gained researchers' attention (Kherif et al., 2011; Wager et al., 2013). Considering that the automatic preattentive processing discovered via the MMN paradigm can be induced by either acoustic properties or long-term language experience, the MMN component is able to reflect not only bottom-up processing, but also top-down processing relevant for semantics and syntax at the preattentive stage (Pulvermüller, 2001; Pulvermüller et al., 2001a,b; Pulvermüller and Shtyrov, 2006; Penolazzi et al., 2007; Shtyrov and Pulvermüller, 2007; Gu et al., 2012). Top-down processing was also found as early as around 200 ms after stimulus onset with attention (Bonte et al., 2006). However, there was an MMN study (Luo et al., 2006) of tone perception only found a general right lateralization of tone perception. Two factors may cause this. First, many of these experiments only concern acoustic processing, i.e., bottom-up processing at the preattentive stage (e.g., Luo et al., 2006). Second, without recruiting linguistic factors, top-down processing that is dominant primarily in the left hemisphere and in the case of semantics processing would have the least influence at the preattentive stage (e.g., Xi et al., 2010).

In our study, we consider both semantic roles that require linguistic top-down processing and pitch variations that require acoustic bottom-up processing in active, language-relevant tasks, which distinguish our experiments from those MMN experiments. Via a series of tasks that involve different levels of language processing, we explicitly address top-down processing at both the early and late stages of lexical tone perception, and gather consistent evidence of the co-occurrence of top-down and bottom-up processing at those stages.

## Language processing and general cognitive functions

Separating lexical tone perception into bottom-up and top-down processing not only decomposes this language-specific function into general cognitive functions like sensory or memory, but also reveals that speech processing share similar mechanisms with other cognitive functions. For example, there are bottom-up attention (automatic attention shift to an unexpected event, without requiring any sort of executive processing nor involving any active engagement beforehand) and task-related top-down attention (Connor et al., 2004; Buschman and Miller, 2007; Pinto et al., 2013), both of which take part in information processing.

On the one hand, although previous work on speech perception focuses mainly on the left hemisphere, there are ample findings arguing against the existence of a centralized “core” in the left hemisphere dedicated exclusively to language processing. For example, the language function of intonation shows a right hemisphere advantage (Gandour et al., 2003b). Following a decompositional view, such right hemisphere advantage can be ascribed to consistent right hemisphere advantages of general cognitive components, including perception of slow-varying cues and emotions. Although lexical tone perception is special in the sense that it involves advantageous components in both the left hemisphere (semantic processing) and the right hemisphere (pitch processing), we can apply the same view to it. Similarly, this decompositional view can also be extended to aspects of semantics and syntax. Rather than arguing that there is no brain region that is specific for language processing, what the decompositional view emphasizes is that language must be supported by many general functions and share or recruit similar computational resources as those general functions.

On the other hand, it is not uncommon to conceptualize complex cognitive functions like language as a combination of general functions in terms of cognitive models and neural circuitry (Dehaene and Cohen, 2007; Hurley, 2007; Anderson, 2010). Take the example of attention, there is a heat debate on whether our attention is drawn voluntarily by top-down, task-dependent factor or involuntarily by bottom-up, saliency factor (Theeuwes, 1991; Buschman and Miller, 2007). Similarly, language processing also involves domain-general functions (Yip, 2002; Hurford, 2007; Fitch, 2010; Arbib, 2012). Although examining top-down and bottom-up mechanisms in cognitive functions is already prevailing, such a separation has not been commonly practiced in previous language processing literature.

Noting these, unlike the previous research that puts too much emphasis on discovering domain-specific cognitive or neural mechanisms for language processing, we advocate that decomposing language functions into more basic components (Fitch, 2010) and locating the neural networks that systematically marshal these functions (Sporns, 2011) can lead to rigorous views about the essential commonalities between language and other cognitive functions.

## Conclusion

In this paper, we reported three ERP experiments that collectively illustrated that both bottom-up processing and top-down processing during lexical tone perception co-occurred in both the early (around 200 ms) and late (around 300–500 ms) time windows of processing. Based on these findings, we proposed a parallel lexical tone processing model that entailed both types of processing throughout various processing stages. This experimental study discussed not only the temporal relation between bottom-up and top-down processing during tone perception, but also the similarities between language processing and other cognitive functions, the latter of which pointed out an important direction in future research of language processing and general cognition.

### Conflict of interest statement

The authors declare that the research was conducted in the absence of any commercial or financial relationships that could be construed as a potential conflict of interest.
